# Thymosin β4 alleviates renal fibrosis and tubular cell apoptosis through TGF-β pathway inhibition in UUO rat models

**DOI:** 10.1186/s12882-017-0708-1

**Published:** 2017-10-18

**Authors:** Jing Yuan, Yan Shen, Xia Yang, Ying Xie, Xin Lin, Wen Zeng, Yingting Zhao, Maolu Tian, Yan Zha

**Affiliations:** Department of Nephrology Guizhou Provincial People’s Hospital, Guiyang, 550002 China

**Keywords:** Thymosin β4, Transforming growth factorβ, Renal fibrosis, Cell apoptosis

## Abstract

**Background:**

Thymosin β4 (Tβ4) is closely associated with the cytoskeleton, inflammation, wound healing, angiogenesis, apoptosis, and myocardial regeneration, but the effects of Tβ4 treatment on chronic renal tubular interstitial fibrosis (CRTIF) are poorly known. This study aimed to examine the effects of Tβ4 on the renal apoptosis and the expression of transforming growth factor (TGF-β), E-cadherin, and α-smooth muscle actin (α-SMA) in CRTIF rat models.

**Methods:**

Male SD rats were randomized into four groups (sham group, unilateral ureteral obstruction (UUO) group, UUO + low-dose Tβ4 group, and UUO + high-dose Tβ4 group). The pathological changes of kidney tissue and its function were assessed two weeks after UUO. In renal interstitial tissue,TGF-β, E-cadherin and α-SMA expression was detected by western blot. In tubular epithelial cells, E-cadherin and α-SMA expression was detected using Real-time qPCR and western blot. Cell apoptosis of rat renal interstitial tissue and tubular epithelial cells was evaluated by immunofluorescence and western blot.

**Results:**

Two weeks after UUO, no differences in blood urea nitrogen and creatinine were observed between the four groups (*P* > 0.05). Compared to the UUO group, Tβ4 treatment decreased the 24-h proteinuria (*P* < 0.001) and reduced the area of pathological change (*P* < 0.01); this effect was more apparent in the UUO + high-dose Tβ4 group. Compared to the UUO group, a significant decrease in TGF-β and α-SMA protein expression was observed in the high-dose Tβ4 group. The level of E-cadherin protein was lower in the UUO group than the Tβ4 groups, and high-dose Tβ4 treatment further increased E-cadherin expression and improved cell apoptosis in the renal interstitial tissue. Analysis of in vitro tubular epithelial cells showed that α-SMA mRNA and protein expression decreased, while E-cadherin mRNA and protein expression increased by Tβ4 treatment. Similarly, these changes were more significant in the UUO + high-dose Tβ4 group. Tβ4 treatment improved the apoptosis of In vitro tubular epithelial cells compared with pure TGF-β stimulation, and equally, the decrease of apoptosis was more apparent in the TGF-β + high-dose Tβ4 group.

**Conclusions:**

Tβ4 treatment might alleviate the renal fibrosis and apoptosis of tubular epithelial cells through TGF-β pathway inhibition in UUO rats with CRTIF.

**Electronic supplementary material:**

The online version of this article (10.1186/s12882-017-0708-1) contains supplementary material, which is available to authorized users.

## Background

Chronic kidney disease (CKD) is characterized by abnormalities of kidney structure or function that are present >3 months and has an impact on the health of the patient [1]. According to recent epidemiological data, the prevalence of CKD in China has reached 10.8%, which means that approximately 119.5 million people currently suffer from CKD in China [2]. Kidney disease is often due to direct injury, yet in many cases, this initial insult will initiate fibrogenesis, especially when healing process was applied by regeneration inadequate [3]. Renal fibrosis is the main pathological change of many CKDs and its pathological features include accumulation of ECM an extension or atrophy of the renal tubule [4].

Transforming growth factor (TGF)-β is widely regarded as a key cytokine promoting fibrosis [5]. TGF-β induces the transformation of tubular epithelial cells and mesangial cells into fibroblast cells, downregulates E-cadherin expression, and upregulates α-smooth muscle actin (SMA) expression [6]. TGF-β can induce fibroblast cells to express α-SMA, accelerating the shrinkage of fibrotic tissues and leading to ischemia and hypoxia of renal tissues [7]. Therefore, inhibiting the expression of fibrosis -related factors such as TGF-β might be a way to control or even reverse renal fibrosis, but there is a lack of reliable and effective drugs.

Thymosin β4 (Tβ4) might be a solution against renal fibrosis. In recent years, a large number of in vivo and in vitro experiments have demonstrated that Tβ4 is a multifunctional protein and has important roles in tissue repair and regeneration [8, 9]. Because of its effects of anti-apoptosis, angiogenesis, anti-inflammation, and promotion of cell migration, Tβ4 plays central roles in promoting wound healing, tissue regeneration, and angiogenesis [10, 11]. Previous studies examined the inhibitory effects of various anti-fibrosis drugs including Tβ4 on fibrosis -related factors in rats with CKD [12, 13], but rare relevant research about the inhibitory effect of Tβ4 on TGF-β has been performed.

Therefore, we hypothesized TGF-β is inhibited by Tβ4, leading to alleviate renal fibrosis and cell apoptosis. This study aimed to examine the effects of different doses of Tβ4 on the expression of TGF-β, E-cadherin, α-SMA and apoptosis-related factors in CRTIF rats (Additional file 1).

## Methods

### Animals

Sixty Sprague Dawley male rats weighing 180–220 g and aged 6–8 weeks of specific pathogen-free grade were provided by the Chongqing Animal Center (permit number scxk-2007-0005). Rats were housed five rats/cage at 23 ± 2 °C and humidity of 55 ± 2%. Food and water intake was recorded daily. The litter was replaced daily. The rats were randomized to the sham group (*n* = 15), unilateral ureteral obstruction (UUO) group (*n* = 15), UUO + low-dose Tβ4 (1 mg/kg•d) group (*n* = 15), and UUO + high-dose Tβ4 (5 mg/kg•d) group (*n* = 15). The UUO and sham groups were given equal volumes of normal saline for intragastric lavage.

The rat model of UUO is currently regarded as the best animal model of progressive chronic renal tubule interstitial fibrosis [14]. The surgery was performed as described in the literature [14]. The rats received an intraperitoneal injection of 10% chloral hydrate (0.3 mL/100 g) and were placed laterally on the right side. Skin preparation and disinfection were performed on the left rib on the back. An incision was made 0.5 cm at the left rib on the back. The abdominal cavity was opened and the left kidney and ureter was located and isolated using blunt dissection. The left ureter was held up with a tissue clamp and nipped with a hemostat at the upper middle segment. After the two ends were ligated, the left ureter between the ligated sutures was cut and removed. The incision was closed by suture layer. In the sham group, the abdominal cavity was opened but no tissue was removed.

### Culture of tubular epithelial cells

Tubular epithelial cells (NRK-52E) were obtained from Nan Fang Medical University and grown in DMEM medium (Promo Cell, Heidelberg, Germany), containing 5 mM glucose and 10% heat-inactivated FCS at 37 °C in 5% CO2. Tubular epithelial cells were stimulated with 2.5 ng/ml TGF-β for 72 h in the TGF-βgroup,1μg/ml Tβ4 and 5μg/ml Tβ4 in the low dose Tβ4 group and high dose Tβ4 group for 48 h after TGF-β treated cells, respectively.

### Sample collection

Blood samples were taken from the carotid artery under anesthesia two weeks after UUO. The serum was separated by centrifugation and was used for detection of blood urea nitrogen (BUN) and creatinine. All rats were sacrificed by cervical dislocation, the left kidney was dissected, and the capsule was peeled off. The kidney was fixed in 10% formaldehyde. Paraffin sections were prepared, routine hematoxylin and eosin (H&E) and periodic acid-Schiff (PAS) staining were performed. The remaining sections were used for immunofluorescence. Remaining kidney tissues were stored at −80 °C.

### Kidney histology and morphology

Paraffin sections were processed for PAS staining. Changes of the renal tubule were observed in a double-blind manner under a light microscope [15, 16]. Three sections were chosen from each rat and ten fields of the renal interstitial tissue (avoiding the glomerulus and large blood vessels) were randomly chosen from each section. The area of the renal interstitial tissue was measured by Image pro-Plus6.0 under 200xmagnification. The area ratio of fibrosis and total interstitial tissue was measured in each field. The mean value was used for analysis.

### Real-time qPCR

Total RNA was prepared from the tubular epithelial cells using RNAEasy minikit (Invitrogen, Carlsbad, CA) and reverse transcribed according to the manufacturer’s instructions. Primers for E-cadherin, α-SMA and β-actin were designed and synthesized based on published sequences of these genes as listed in Table 1. Real-time qPCR was performed using SYBR Green PCR Master Mix (Toyobo, Osaka, Japan) and a Rotor- Gene-3000A Real-time PCR System (Corbett, Sydney, Australia) according to the manufacturer’s protocol. The target gene expression was normalized to α β-actin mRNA expression and presented as fold-change compared to the control experiments.

**Table 1:** Primer sequences, product size and annealing temperatures used in RT-PCR

Gene			Product size (pb)	Temperature (°C)
β-actin	Right	GTTGTCGACGACGAGCG	93	60
	Left	GCACAGAGCCTCGCCTT		50
E-cadherin	Right	GACCGGTGCAATCTTCAAA	93	59
	Left	TTGACGCCGAGAGCTACAC		60
α-SMA	Right	GCGTGATTTCCAGCACATAA	101	59
	Left	ATACTTGACCGGGGTCATCC		60

### Western blot

Kidney tissues were placed in radio-immunoprecipitation assay (RIPA) buffer on ice, fully mixed, incubated for 30 min, and centrifuged at 12,000 rpm at 4 °C for 20 min. The supernatant was taken for protein measurement using the bicinchoninic acid assay (BCA) protein detection kit (Sigma, St Louis, Mo, USA). S**odium dodecyl sulfate**(**SDS**) sample buffer (125 mM Tris-HCl pH 6.8, 4% SDS, 20% glycerol, 100 mM dithiothreitol and 0.2% bromophenol blue) was added. The sample was heated at 100 °C for 5 min and stored at −20 °C.

Proteins (50 μg) were separated using 8% or 10% SDS-polyacrylamide gels, and the proteins were transferred to nitrocellulose membranes (Amersham, GE Healthcare, Waukesha, WI, USA). The membranes were incubated with antibodies against E-cadherin (1:2000; GIBCO, Invitrogen Inc., Carlsbad, CA, USA), TGF-β (1:3000 GIBCO, Invitrogen Inc., Carlsbad, CA, USA), α-SMA (1:2000; GIBCO, Invitrogen Inc., Carlsbad, CA, USA),Cleaved Caspase 3 (1:1000, Abcam, UK), Bax(1:1000, Abcam, UK), and Bcl-2 antibodies (1:1500, Abcam, UK) in tris-buffered saline-tween 20 (TBST) containing 2% BSA overnight at 4 °C. After washing, membranes were incubated with horseradish peroxidase-labeled secondary antibody (1:1000;GIBCO, Invitrogen Inc., Carlsbad, CA, USA) in TBST containing 2% BSA at room temperature for 1 h. Enhanced chemiluminescence (Amersham, GE Healthcare, Waukesha, WI, USA) was used for detection. Signal intensity was analyzed using the ScionImage gel image analysis system (Scion Corporation, Frederick, MD, USA). β-actin was used as internal control. Quantitative analysis of the western blot bands was conducted using the Labwork analysis software. The 15 samples per group was detected.

### Apoptotic determination

TUNEL staining was carried out using a promega apoptosis detection kit. Immunofluorescence for TUNEL staining was performed with Alexa Fluor 594-conjugated goat anti-mouse IgG (1: 500; Invitrogen). The glass was mounted with cover slips containing Vectashield mounting medium with 4′,6-diamidino-2-phenylindole (DAPI; Sigma) and imaged under an fluorescent microscope.

### Statistical analysis

SPSS16.0 (IBM, Armonk, NY, USA) was used for statistical analysis. Continuous data are expressed as mean ± standard deviation (SD) and were analyzed using one-way ANOVA followed by the LSD post hoc test. Two-sided *P*-values <0.05 were considered statistically significant. The data was analyzed in normally distributed by Kolmogorov-Smirnov (*P* > 0.05), data obeyed the normal distribution.

## Results

### Effects of Tβ4 on kidney functions and area of renal interstitial fibrosis in the rat model of UUO

Proteinuria was reduced in the Tβ4 groups (*P* < 0.01), and the decrease was more obvious in the UUO + high-dose Tβ4 group (*P* < 0.05). No difference was found in kidney function (*P* > 0.05), but pathological changes in the renal tubulointerstitium was significantly reduced (*P* < 0.05), particularly in the high-dose group (Table 2).

**Table 2:** Changes of kidney function and area ratio of renal interstitial fibrosis in different groups

	Sham	UUO	UUO + low-dose Tβ4	UUO + high-dose Tβ4
Proteinuria (mg/24 h)	13.32 ± 4.19	251.30 ± 28.63^△^	101.31 ± 19.04^#^	69.25 ± 10.27^▲^
BUN (mmol/L)	6.31 ± 1.02	9.24 ± 2.31	7.82 ± 1.95	7.07 ± 1.67
Creatinine (mmol/L)	50.35 ± 3.78	81.24 ± 12.54	69.82 ± 13.68	64.73 ± 14.06
Area ratio of fibrosis and total interstitial tissue	0.053 ± 0.008	0.55 ± 0.04^△^	0.31 ± 0.05^#^	0.074 ± 0.006^*^

△ *P* < 0.001 vs. the sham group

# *P* < 0.01 vs. the UUO group

▲*P* < 0.01, * *P* < 0.05 vs. the UUO + low-dose Tβ4 group

*UUO* unilateral ureteral obstruction*, Tβ4* thymosin β4, *BUN* blood urea nitrogen

### Effect of different doses of Tβ4 on histology and morphology of kidney tissue in the rat model of UUO

**Fig. 1:**
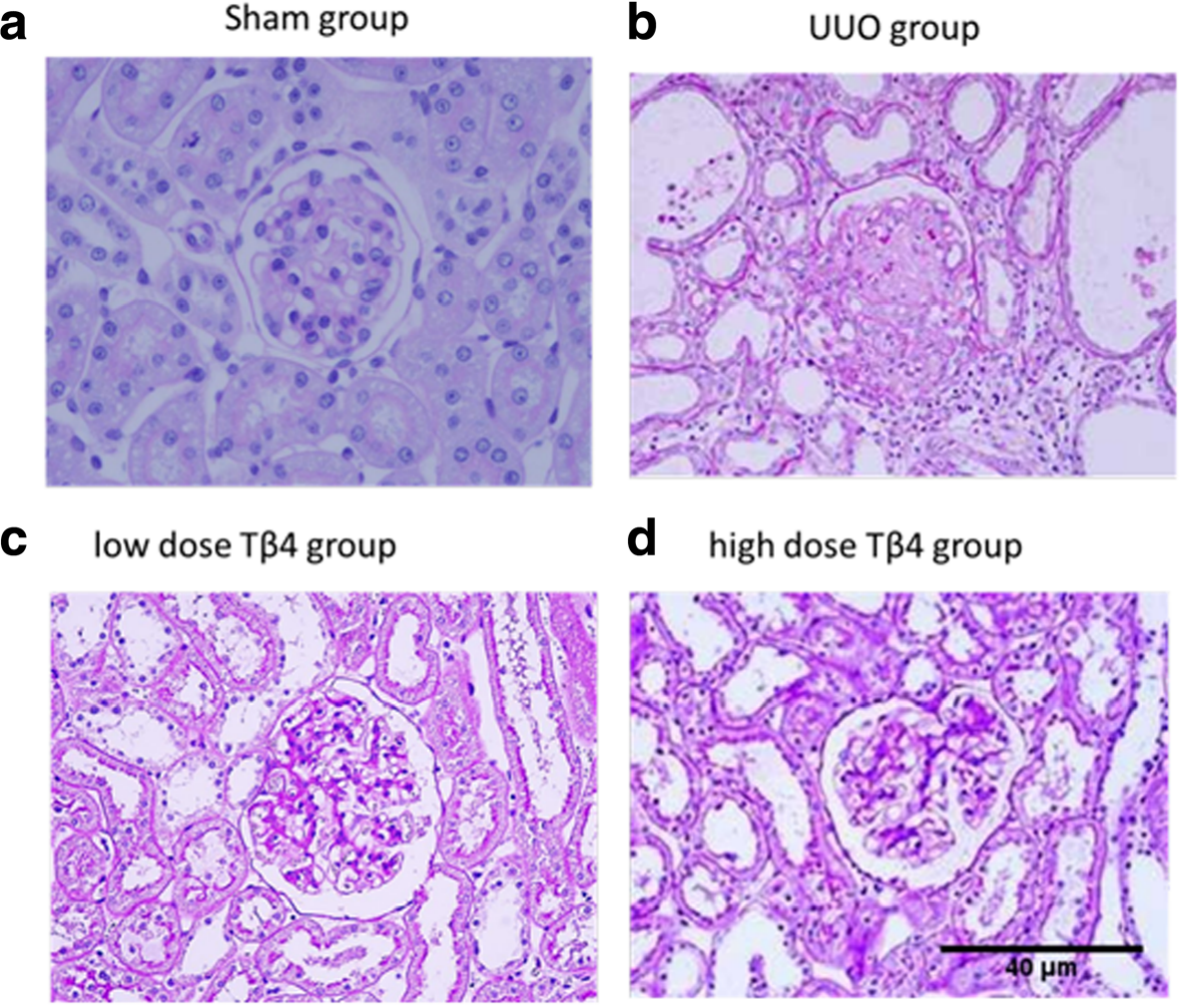
Effects of different doses of Tβ4 on the pathological changes in kidney tissue in a rat model of UUO. **a** In the sham group, regular arrangement of tissue structures of the kidney with normal glomerulus and renal tubule. **b** In the UUO group, severe atrophy of the glomerulus, diffusely widened capsule area, enlarged tubular cavity, vacuolar degeneration, and swollen epithelial cells in the proximal convoluted tubule around the medullary loop, exfoliated epithelial cells in the area with severe pathological changes, disrupted and atrophy renal interstitial tissue, and fibrosis were observed. **c** The pathological damage was alleviated in the UUO + low-dose Tβ4 group. **d** The damages were further alleviated in the UUO + high-dose Tβ4 group. Periodic Acid-Schiff staining ×200 magnification

Normal morphology of kidney tissue is the base of kidney function. In the sham group, organization of kidney tissue was in good order and no abnormal pathological changes were observed in the glomerulus and renal tubule (Fig. 1a). In the UUO group, severe atrophy was seen in the glomerulus, the capsule area was diffusely widened, the tubular cavity was enlarged, and the epithelial cells in the proximal convoluted tubule around the medullary loop showed vacuolar degeneration and were swollen. In the area with severe pathological changes, exfoliated epithelial cells were observed and the renal interstitial tissue was disrupted and atrophied, and had a tendency for fibrosis (Fig. 1b). Pathological damage was alleviated to a greater extent in the UUO + high-dose Tβ4 group (Fig. 1d) compared to the UUO + low-dose Tβ4 group (Fig. 1c).

### Detection of TGF-β expression in the rat kidney tissue by western blot

**Fig. 2:**
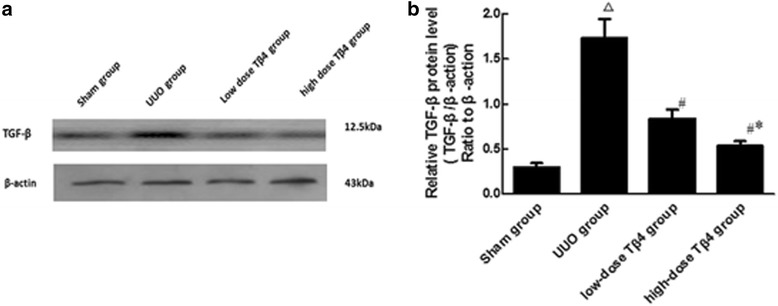
Effects of different doses of Tβ4 on TGF-β expression in renal tubulointerstitial tissue of UUO rat model. **a** Western blot. **b** Quantification of A. ^△^ *P* < 0.01 vs. the sham group, ^#^ *P* < 0.05 vs. the UUO group, ^*^ *P* < 0.05 vs. the UUO + low-dose Tβ4 group

TGF-β protein expression in the UUO group was markedly increased compared to the sham group (Fig. 2a-b; *P* < 0.01). The expression was significantly decreased in the low-dose Tβ4 group compared to the model group (Fig. 2a-b) (*P* < 0.05), and the expression was even decreased to a greater extent in the high-dose Tβ4 group (Fig. 2a-b) (*P* < 0.05).

### Detection of E-cadherin and α-SMA expressions in kidney tissue from different groups by western blot

**Fig. 3:**
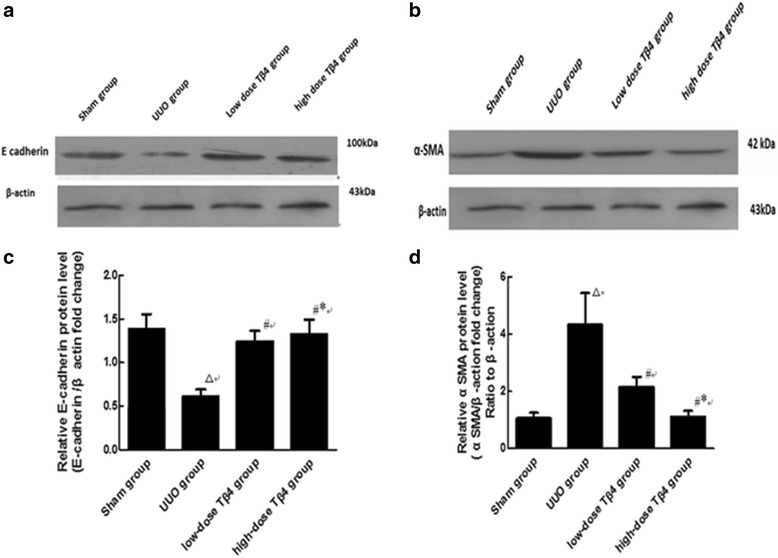
Effects of Tβ4 on the E-cadherin and α-SMA levels in the renal tubulointerstitial tissue of UUO rat model. **a** and **b** Western blot. **c** and **d** Quantification of A.^△^ *P* < 0.01 vs. the sham group; ^#^ *P* < 0.05 vs. the UUO group; ^*^ *P* < 0.05 vs. the UUO + low-dose Tβ4 group

E-cadherin expression in the UUO group was significantly lower compared to the sham group (Fig. 3a, c) (*P* < 0.01). The expression of E-cadherin in the UUO + low-dose group was markedly increased compared to the UUO group (*P* < 0.05). The increase in E-cadherin expression was greater in the high-dose group compared to the low-dose group (Fig. 3a, c) (*P* < 0.05). α-SMA expression in the UUO group was significantly elevated compared to the sham group (Fig. 3b, d) (*P* < 0.01). The expression of α-SMA in the UUO + low-dose group was markedly decreased (*P* < 0.05). The reduction in α-SMA expression was greater in the UUO + high-dose group compared to the UUO + low-dose group (Fig. 3b, d) (*P* < 0.05).

### Effects of different doses of Tβ4 on E-cadherin and a-SMA in TGF-β induced renal tubular cells

**Fig. 4:**
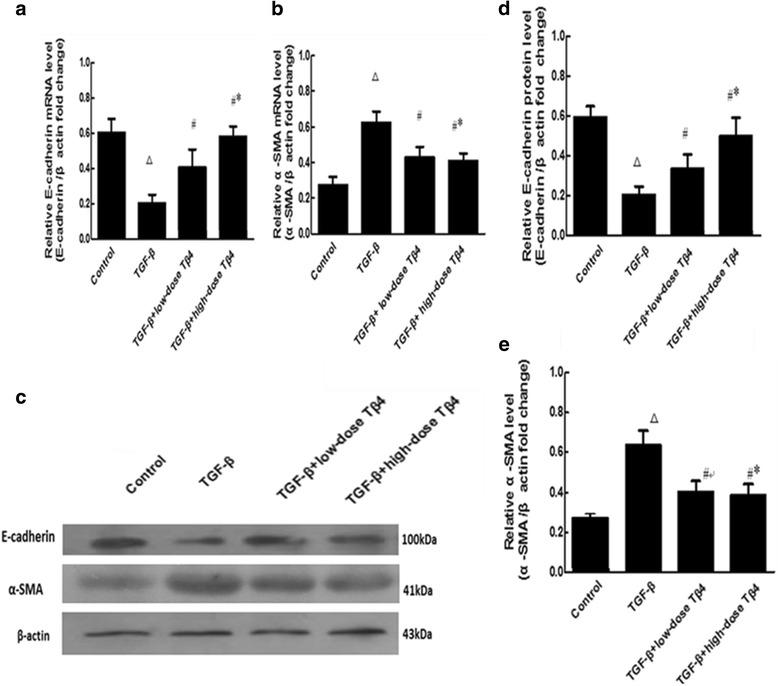
Effects of different doses of Tβ4 on E-cadherin and a-SMA in TGF-β induced renal tubular cells. **a** and **b** mRNAs of E-cadherin and a-SMA were detected by qPCR. **c** E-cadherin and a-SMA proteins were detected by Western blot. **d** and **e** Quantification of C. ^△^ *P* < 0.01 vs. the control group; ^#^ *P* < 0.05 vs. the TGF-β group; ^*^ *P* < 0.05 vs. The TGF-β + low-dose Tβ4. *n* = 4

*E-cadherin* mRNA in the TGF-β group was significantly lower compared to the control group (Fig. 4a, P<0.01), the expression of *E-cadherin* mRNA in the TGF-β+low-dose Tβ4 group was markedly increased compared to the TGF-β group (Fig. 4a, P<0.05). The increase in *E-cadherin* mRNA expression was greater in the TGF-β+high-dose Tβ4 group compared to the low-dose group (Fig. 4a, P<0.05). 훼-SMA mRNA expression in the TGF-β group was significantly elevated compared to the control group (Fig. 4b *P* < 0.01). The expression of *α-SMA* mRNA in the TGF-β + low-dose Tβ4 group was markedly decreased (Fig. 4b *P* < 0.05). The reduction in α-SMA expression was greater in the TGF-β + high-dose Tβ4 group compared to the low-dose group (Fig. 4b *P* < 0.05).

E-cadherin expression in the TGF-β group was significantly lower compared to the control group (Fig. 4c, d) (*P* < 0.01). The expression of E-cadherin in the TGF-β + low-dose Tβ4 group was markedly increased compared to the TGF-β group (*P* < 0.05). The increase in E-cadherin expression was greater in the TGF-β + high-dose Tβ4 group compared to the low-dose group (Fig. 4c, d) (*P* < 0.05). α-SMA expression in the TGF-β group was significantly elevated compared to the control group (Fig. 4c, e) (*P* < 0.01). The expression of α-SMA in the TGF-β + low-dose Tβ4 group was markedly decreased (*P* < 0.05). The reduction in α-SMA expression was greater in the TGF-β + high-dose Tβ4 group compared to the low-dose group (Fig. 4c, e) (*P* < 0.05).

### Effects of different doses of Tβ4 on renal tubular cells apoptosis in the UUO rat models

**Fig. 5:**
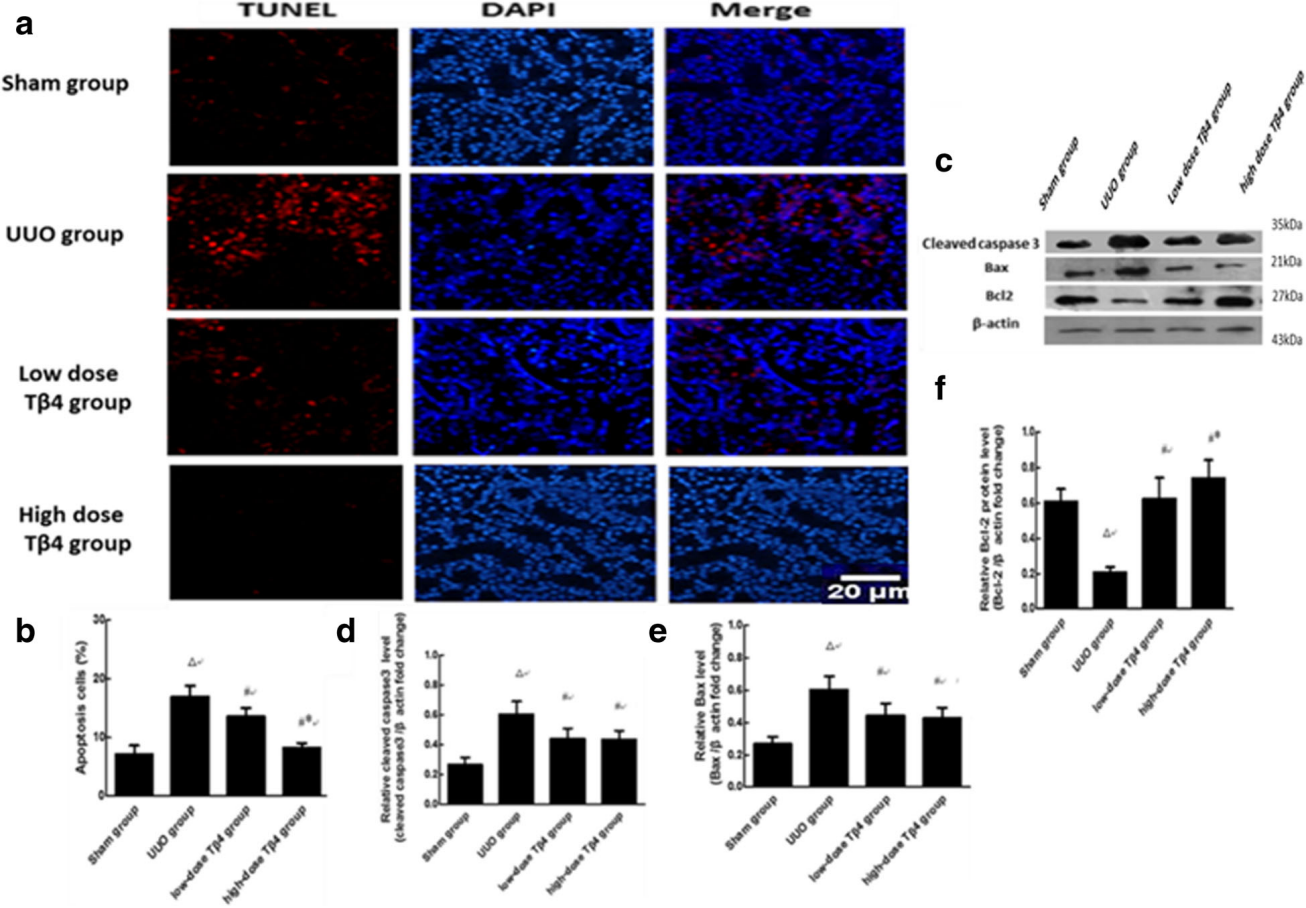
Tβ4 attenuates renal tubular cells apoptosis in the rat model of UUO. **a** Representative microphotographs of TUNEL staining in kidney sections. TUNEL is stained in red and nuclei are labeled by DAPI staining in blue. The sections are shown at an original magnification of 400. **b** Representative immunoblotting of cleaved caspase-3, Bax and Bcl-2. **c**, **d** and **e** Quantification of western blot. ^△^ *P* < 0.01 vs. the sham group; ^#^ *P* < 0.05 vs. the UUO group; ^*^ *P* < 0.05 vs. the UUO+ low-dose Tβ4 group

TUNEL staining of kidney sections in the UUO group was significantly strengthened compared to the sham group (Fig. 5a, b) (*P* < 0.01). The TUNEL staining in the low-dose Tβ4 group was markedly weakened (*P* < 0.05). The reduction in TUNEL staining was greater in the high-dose Tβ4 group compared to the low-dose group (Fig. 5a, b) (*P* < 0.05).

Significantly decreased Bcl-2 in the UUO group compared with sham group was found (Fig. 5c, d) (*P* < 0.01). The expression of Bcl-2 in the low-dose Tβ4 group was markedly increased compared to the UUO group (*P* < 0.05). The increase in Bcl-2 expression was greater in the high-dose Tβ4 group compared to the low-dose group (Fig. 5c, f) (*P* < 0.05). The expressions of Bax and cleaved caspase-3 in the UUO group were significantly elevated compared to the sham group (Fig. 5c–e) (*P* < 0.01). Compared with UUO group, the expressions of Bax and cleaved caspase-3 in the low-dose and high-dose Tβ4 groups were markedly decreased (*P* < 0.05).

### Tβ4 treatment attenuated apoptosis induced by TGF-β in renal tubular cells

**Fig. 6:**
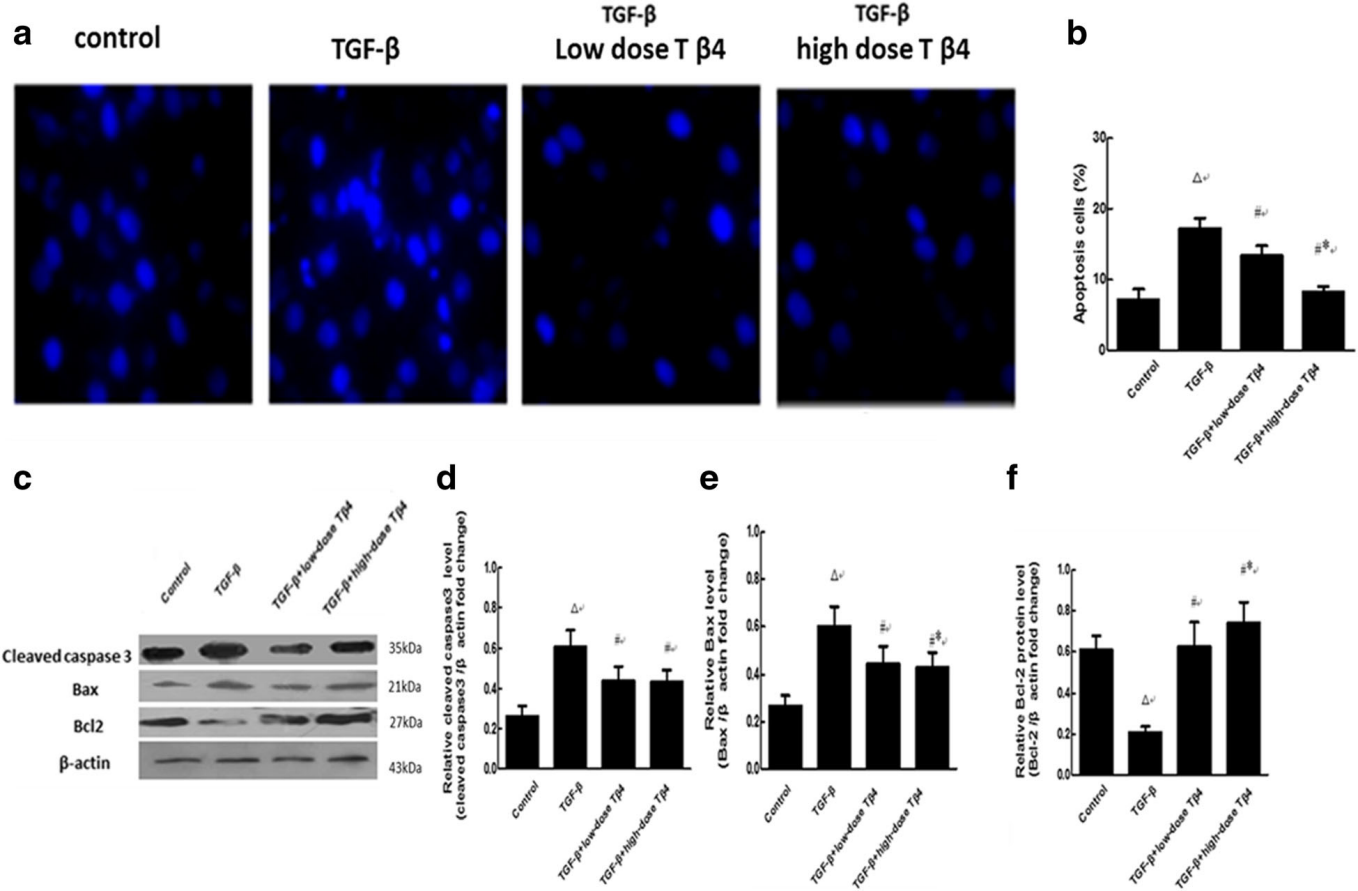
Tβ4 attenuated apoptosis induced by TGF-βin renal tubular cells. **a** and **b** Observations of morphological alterations of apoptosis cells were addressed by Hoechst33258 staining and counted by Image J software; **c** Cleaved caspase-3, Bax and Bcl-2 protein levels were determined by western blotting in renal tubular cells. **d**, **e** and **f** Quantification of western blotting. ^△^ *P* < 0.01 vs. control; ^#^ *P* < 0.05 vs. the TGF-βgroup; ^*^ *P* < 0.05 vs. the TGF-β + low-dose Tβ4 group. *n* = 4

Observations of morphological alterations of apoptosis cells were addressed by tunnel staining and counted by Image J software. The number of apoptosis cells in the TGF-β group was significantly elevated compared to the control group (Fig. 6a, b *P* < 0.01). The number of apoptosis cells in the TGF-β + low-dose Tβ4 group was markedly decreased (Fig. 6a, b *P* < 0.05). The reduction in apoptosis cells was greater in the TGF-β + high-dose Tβ4 group compared to the low-dose group (Fig. 6a, b *P* < 0.05).

The cleaved caspase-3 and Bax expressions in the TGF-β group were significantly elevated compared to the control group (Fig. 6c–e *P* < 0.01). The expressions of cleaved caspase-3 and Bax in the TGF-β + low-dose or high-dose Tβ4 group were markedly decreased (Fig. 6c–e *P* < 0.05). The reduction in Bax was greater in the TGF-β + high-dose Tβ4 group compared to the low-dose group (Fig. 6c, e *P* < 0.05). Bcl-2 expression in the TGF-β group was significantly lower compared to the control group (Fig. 6c, f) (*P* < 0.01). The expression of Bcl-2 in the TGF-β + low-dose Tβ4 group was markedly increased compared to the TGF-β group (*P* < 0.05). The increase in Bcl-2 expression was greater in the TGF-β + high-dose Tβ4 group compared to the low-dose group (Fig. 6c, f) (*P* < 0.05).

## Discussions

Thymosin is a lymphokine produced by the thymus and was first isolated from calf thymic proteins by Goldstein and White in 1966. Thymosins are small polypeptides containing more than 40 components [4]. Tβ4 has the widest distribution and accounts for 70–80% of total thymosins [17–19]. Currently, synthetic Tβ4 has been used in experiments to explore the mechanisms underlying physiological and pathological activities [20–22]. The inhibitory effects of Tβ4 on some fibrosis factors in rats with progressive chronic renal fibrosis have been examined [12, 13, 23], but few study reported the inhibitory effects of Tβ4 on TGF-β. Therefore, this study aimed to examine the effects of different doses of Tβ4 on CRTIF and on the expression of TGF-β, E-cadherin, and α-SMA.

Results of this showed that two weeks after UUO, there were no differences in kidney function among the four groups. Compared to the UUO group, Tβ4 treatment decreased the 24-h proteinuria, reduced the area of pathological change, and decreased the α-SMA expression. Compared to the UUO group, lower levels of TGF-β protein expression were observed in UUO rats when different doses of Tβ4 were used for treatment. The levels of E-cadherin protein were lower in the UUO group. The above changes were more obvious in the high-dose Tβ4 group than in low- dose Tβ4 group. Cell apoptosis in the renal tissue was improved by Tβ4 treatment in vivo. The results of tubular epithelial cells stimulated by TGF-β shows that α-SMA mRNA and protein levels decreased, E-cadherin mRNA and protein levels increased through Tβ4 treatment, and similarly, these changes were more significant in the TGF-β + high-dose Tβ4 group. The apoptosis of tubular epithelial cells was improved by Tβ4 treatment compared with pure TGF-β stimulation, and equally, the decrease of apoptosis was more obvious in the TGF-β + high-dose Tβ4 group in vitro. These results suggest that Tβ4 treatment might alleviate the kidney fibrosis and apoptosis of tubular epithelial cells by TGF-β pathway inhibition in rats with CKD.

Renal tubulointerstitial fibrosis is a common feature of CKD and a main determinant of progression of renal diseases [4, 12]. The UUO model is an experimental model of renal disease for renal tubulointerstitial fibrosis, chronic inflammation, and interstitial fibrosis caused by continuous ureteral obstruction that can lead to progressive loss of renal function [16]. During the process of fibrosis, over-secretion of TGF-β is regarded as a key factor promoting fibrosis [5]. TGF-β also promotes the transformation of epithelial cells in the renal tubules and capsule cells into fibroblasts, reducing E-cadherin expression and upregulating α-SMA expression [6]. TGF-β can induce fibroblasts cells to express α-SMA, increasing the shrinkage of the fibrosis lesions, consequently leading to ischemia [24]. In this study, the UUO group showed histopathological lesions characteristic of kidney fibrosis, as well as high expression of TGF-β and α-SMA.E-cadherin is a transmembrane glycoprotein maintaining the polarity of epithelial cells and establishing tight junctions. Lower levels of E-cadherin result in the separation of the epithelial cells, changes of phenotypes, and/or cell apoptosis. Lower levels of E-cadherin also reflect the early changes observed during epithelial-mesenchymal transition(EMT) [25, 26]. In this study, E-cadherin expression was decreased in the UUO model group compared to the sham group, but Tβ4 treatment increased the expression of E-cadherin, suggesting decreased EMT and alleviation of kidney fibrosis. In this study, different doses of Tβ4 decreased alleviated kidney fibrosis, suggesting that Tβ4 could antagonize the expression of TGF-β and α-SMA, inhibit cell apoptosis, and increase E-cadherin expression, so reduce EMT. Additional comprehensive studies will be necessary to confirm the mechanism.

## Conclusions

In conclusion, this study suggests that Tβ4 treatment might alleviate the kidney fibrosis and apoptosis of tubular epithelial cells through TGF-β pathway inhibition in rats with CRTIF.Tβ4 could be a novel way to treat CKD, but additional studies are still necessary before clinical trials can be performed.

## Additional file

Additional file 1: Figure S1. Effects of Tβ4 alone on the E-cadherin, α-SMA,cleaved caspase 3,Bax and Bcl2 levels in the renal tubular cells. (A) Western blot.(B) Quantification of A. The results between control group and Tβ4 treated group had no significant difference. (PNG 40 kb)
