# Supplementary figures and images for: Thymosin β4 alleviates renal fibrosis and tubular cell apoptosis through TGF-β pathway inhibition in UUO rat models
Source: BMC Nephrol. 2017 Oct 18;18:314. doi: 10.1186/s12882-017-0708-1 (PMC5648500; doi:10.1186/s12882-017-0708-1)

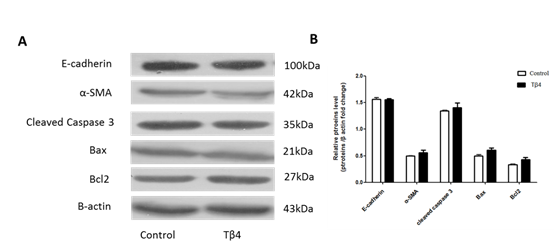

Supplement: Additional file 1: Figure S1. — Effects of Tβ4 alone on the E-cadherin, α-SMA,cleaved caspase 3,Bax and Bcl2 levels in the renal tubular cells. (A) Western blot.(B) Quantification of A. The results between control group and Tβ4 treated group had no significant difference. (PNG 40 kb) [file 12882_2017_708_MOESM1_ESM.png]
